# Data Management for the Internet of Things: Design Primitives and Solution

**DOI:** 10.3390/s131115582

**Published:** 2013-11-14

**Authors:** Mervat Abu-Elkheir, Mohammad Hayajneh, Najah Abu Ali

**Affiliations:** 1 Faculty of Computer and Information Sciences, Mansoura University, Mansoura 35516, Egypt; 2 College of Information Technology, United Arab Emirates University, Al-Ain 17551, Abu Dhabi; E-Mails: mhayajneh@uaeu.ac.ae (M.H.); najah@uaeu.ac.ae (N.A.A.)

**Keywords:** Internet of Things, data management, sensor networks

## Abstract

The Internet of Things (IoT) is a networking paradigm where interconnected, smart objects continuously generate data and transmit it over the Internet. Much of the IoT initiatives are geared towards manufacturing low-cost and energy-efficient hardware for these objects, as well as the communication technologies that provide objects interconnectivity. However, the solutions to manage and utilize the massive volume of data produced by these objects are yet to mature. Traditional database management solutions fall short in satisfying the sophisticated application needs of an IoT network that has a truly global-scale. Current solutions for IoT data management address partial aspects of the IoT environment with special focus on sensor networks. In this paper, we survey the data management solutions that are proposed for IoT or subsystems of the IoT. We highlight the distinctive design primitives that we believe should be addressed in an IoT data management solution, and discuss how they are approached by the proposed solutions. We finally propose a data management framework for IoT that takes into consideration the discussed design elements and acts as a seed to a comprehensive IoT data management solution. The framework we propose adapts a federated, data- and sources-centric approach to link the diverse Things with their abundance of data to the potential applications and services that are envisioned for IoT.

## 1. Introduction

The IoT is a dynamic and global network infrastructure, in which “Things”—subsystems and individual physical and virtual entities—are identifiable, autonomous, and self-configurable. “Things” are expected to communicate among themselves and interact with the environment by exchanging data generated by sensing, while reacting to events and triggering actions to control the physical world [1]. The vision that the IoT should strive to achieve is to provide a standard platform for developing cooperative services and applications that harness the collective power of resources available through the individual “Things” and any subsystems designed to manage the aforementioned “Things”. At the center of these resources is the wealth of information that can be made available through the fusion of data that is produced in real-time as well as data stored in permanent repositories. This information can make the realization of innovative and unconventional applications and value-added services possible, and will provide an invaluable source for trend analysis and strategic opportunities. A comprehensive management framework of data that is generated and stored by the objects within IoT is thus needed to achieve this goal.

Data management is a broad concept referring to the architectures, practices, and procedures for proper management of the data lifecycle needs of a certain system. In the context of IoT, data management should act as a layer between the objects and devices generating the data and the applications accessing the data for analysis purposes and services. The devices themselves can be arranged into subsystems or subspaces with autonomous governance and internal hierarchical management [2]. The functionality and data provided by these subsystems is to be made available to the IoT network, depending on the level of privacy desired by the subsystem owners.

IoT data has distinctive characteristics that make traditional relational-based database management an obsolete solution. A massive volume of heterogeneous, streaming and geographically-dispersed real-time data will be created by millions of diverse devices periodically sending observations about certain monitored phenomena or reporting the occurrence of certain or abnormal events of interest [3]. Periodic observations are most demanding in terms of communication overhead and storage due to their streaming and continuous nature, while events present time-strain with end-to-end response times depending on the urgency of the response required for the event. Furthermore, there is metadata that describes “Things” in addition to the data that is generated by “Things”; object identification, location, processes and services provided are an example of such data. IoT data will statically reside in fixed- or flexible-schema databases and roam the network from dynamic and mobile objects to concentration storage points. This will continue until it reaches centralized data stores. Communication, storage and process will thus be defining factors in the design of data management solutions for IoT.

It has been projected recently that there is a renewed interest in database systems research that focuses on alternate models other than the traditional relational model. The shift from traditional database models has a number of aspects that are especially useful to IoT, such as the utilization of remote storage at the Things layer, non-structural data support, relaxation of the Atomicity, Consistency, Isolation, and Durability (ACID) properties to trade-off consistency and availability, and integration of energy efficiency as a data management design primitive [4].

In this paper, we highlight the data management lifecycle from the perspective of IoT architecture and show why it should be different from traditional data management systems. Both offline and real-time data cycles need to be supported in an IoT-based data management system, to accommodate the various data and processing needs of potential IoT users. We subsequently review the work that has been done in data management for IoT and its potential subsystems and analyze the current proposals against a set of proposed design elements that we deem necessary in IoT data management solutions.

A data management framework for IoT is presented that incorporates a layered, data-centric, and federated paradigm to join the independent IoT subsystems in an adaptable, flexible, and seamless data network. In this framework, the “Things” layer is composed of all entities and subsystems that can generate data. Raw data, or simple aggregates, are then transported via a communications layer to data repositories. These data repositories are either owned by organizations or public, and they can be located at specialized servers or on the cloud. Organizations or individual users have access to these repositories via query and federation layers that process queries and analysis tasks, decide which repositories hold the needed data, and negotiate participation to acquire the data. In addition, real-time or context-aware queries are handled through the federation layer via a sources layer that seamlessly handles the discovery and engagement of data sources. The whole framework therefore allows a two-way publishing and querying of data. This allows the system to respond to the immediate data and processing requests of the end users and provides archival capabilities for later long-term analysis and exploration of value-added trends.

The rest of this paper is organized as follows: Section 2 discusses IoT data management and describes the lifecycle of data within IoT. Section 3 discusses the current approaches to IoT data management, and gives an analysis of how they satisfy a set of design elements that should be considered. In Section 4, a data management framework for IoT is proposed and its components are outlined. Finally, Section 5 concludes the paper.

## 2. IoT Data Management

Traditional data management systems handle the storage, retrieval, and update of elementary data items, records and files. In the context of IoT, data management systems must summarize data online while providing storage, logging, and auditing facilities for offline analysis. This expands the concept of data management from offline storage, query processing, and transaction management operations into online-offline communication/storage dual operations. We first define the data lifecycle within the context of IoT and then outline the energy consumption profile for each of the phases in order to have a better understanding of IoT data management.

### 2.1. IoT Data Lifecycle

**Figure 1.**
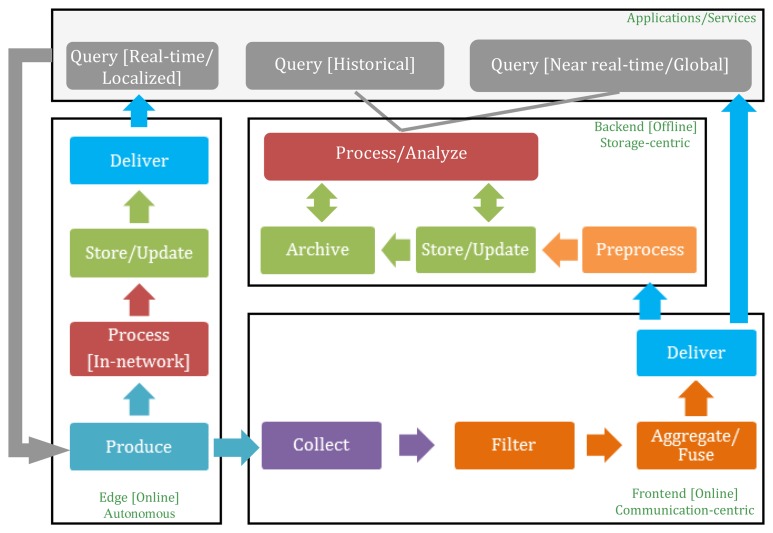
IoT data lifecycle and data management.

The lifecycle of data within an IoT system—illustrated in Figure 1—proceeds from data production to aggregation, transfer, optional filtering and preprocessing, and finally to storage and archiving. Querying and analysis are the end points that initiate (request) and consume data production, but data production can be set to be “pushed” to the IoT consuming services [5]. Production, collection, aggregation, filtering, and some basic querying and preliminary processing functionalities are considered online, communication-intensive operations. Intensive preprocessing, long-term storage and archival and in-depth processing/analysis are considered offline storage-intensive operations.

Storage operations aim at making data available on the long term for constant access/updates, while archival is concerned with read-only data. Since some IoT systems may generate, process, and store data in-network for real-time and localized services, with no need to propagate this data further up to concentration points in the system, “edges” that combine both processing and storage elements may exist as autonomous units in the cycle. In the following paragraphs, each of the elements in the IoT data lifecycle is explained.

*Querying*: Data-intensive systems rely on querying as the core process to access and retrieve data. In the context of IoT, a query can be issued either to request real-time data to be collected for temporal monitoring purposes or to retrieve a certain view of the data stored within the system. The first case is typical when a (mostly localized) real-time request for data is needed. The second case represents more globalized views of data and in-depth analysis of trends and patterns.

*Production*: Data production involves sensing and transfer of data by the “Things” within the IoT framework and reporting this data to interested parties periodically (as in a subscribe/notify model), pushing it up the network to aggregation points and subsequently to database servers, or sending it as a response triggered by queries that request the data from sensors and smart objects. Data is usually time-stamped and possibly geo-stamped, and can be in the form of simple key-value pairs, or it may contain rich audio/image/video content, with varying degrees of complexity in-between.

*Collection:* The sensors and smart objects within the IoT may store the data for a certain time interval or report it to governing components. Data may be collected at concentration points or gateways within the network where it is further filtered and processed, and possibly fused into compact forms for efficient transmission. Wireless communication technologies such as Zigbee, Wi-Fi and cellular are used by objects to send data to collection points.

*Aggregation/Fusion*: Transmitting all the raw data out of the network in real-time is often prohibitively expensive given the increasing data streaming rates and the limited bandwidth. Aggregation and fusion techniques deploy summarization and merging operations in real-time to compress the volume of data to be stored and transmitted [6].

*Delivery*: As data is filtered, aggregated, and possibly processed either at the concentration points or at the autonomous virtual units within the IoT, the results of these processes may need to be sent further up the system, either as final responses, or for storage and in-depth analysis. Wired or wireless broadband communications may be used there to transfer data to permanent data stores.

*Preprocessing*: IoT data will come from different sources with varying formats and structures. Data may need to be preprocessed to handle missing data, remove redundancies and integrate data from different sources into a unified schema before being committed to storage. This preprocessing is a known procedure in data mining called data cleaning. Schema integration does not imply brute-force fitting of all the data into a fixed relational (tables) schema, but rather a more abstract definition of a consistent way to access the data without having to customize access for each source's data format(s). Probabilities at different levels in the schema may be added at this phase to IoT data items in order to handle uncertainty that may be present in data or to deal with the lack of trust that may exist in data sources [7].

*Storage/Update—Archiving*: This phase handles the efficient storage and organization of data as well as the continuous update of data with new information as it becomes available. Archiving refers to the offline long-term storage of data that is not immediately needed for the system's ongoing operations. The core of centralized storage is the deployment of storage structures that adapt to the various data types and the frequency of data capture. Relational database management systems are a popular choice that involves the organization of data into a table schema with predefined interrelationships and metadata for efficient retrieval at later stages [8]. NoSQL key-value stores are gaining popularity as storage technologies for their support of big data storage with no reliance on relational schema or strong consistency requirements typical of relational database systems [9]. Storage can also be decentralized for autonomous IoT systems, where data is kept at the objects that generate it and is not sent up the system. However, due to the limited capabilities of such objects, storage capacity remains limited in comparison to the centralized storage model.

*Processing/Analysis*: This phase involves the ongoing retrieval and analysis operations performed and stored and archived data in order to gain insights into historical data and predict future trends, or to detect abnormalities in the data that may trigger further investigation or action. Task-specific preprocessing may be needed to filter and clean data before meaningful operations take place. When an IoT subsystem is autonomous and does not require permanent storage of its data, but rather keeps the processing and storage in the network, then in-network processing may be performed in response to real-time or localized queries.

Looking back at Figure 1, the flow of data may take one of three paths: a path for autonomous systems within the IoT that proceeds from query to production to in-network processing and then delivery, a path that starts from production and proceeds to collection and filtering/aggregation/fusion and ends with data delivery to initiating (possibly global or near real-time) queries, and finally a path that extends the production to aggregation further and includes preprocessing, permanent data storage and archival, and in-depth processing and analysis. In the next section, the need for data management solutions that surpass the current capabilities of traditional data management is highlighted in light of the previously outlined life cycle.

### 2.2. IoT Data Management versus Traditional Database Management Systems

Based on the IoT data lifecycle discussed earlier, we divide an IoT data management system into an online, real-time frontend that interacts directly with the interconnected IoT objects and sensors, and an offline backend that handles the mass storage and in-depth analysis of IoT data. The data management frontend is communication-intensive; involving the propagation of query requests and result to and from sensors and smart objects. The backend is storage-intensive; involving the mass storage of produced data for later processing and analysis and more in-depth queries. Although the storage elements reside on the back end, they interact with the frontend on a frequent basis via continuous updates and are thus referred to as online. The autonomous edges in the life cycle can be considered more communication-intensive than storage-intensive, as they provide real-time data to certain queries.

This envisioned data management architecture parts considerably from the existing database management systems, which are mainly storage-centric. In traditional database systems, the bulk of data is collected from predefined and finite sources, and stored in scalar form according to strict normalization rules in relations. Queries are used to retrieve specific “summary” views of the system or update specific items in the database. New data is inserted into the database when needed, also via insertion queries. Query operations are usually local, with execution costs bound to processing and intermediate storage. Transaction management mechanisms guarantee the ACID properties in order to enforce overall data integrity [8]. Even if the database is distributed over multiple sites, query processing and distributed transaction management are enforced [10]. The execution of distributed queries is based on the transparency principle, which dictates that the database is still viewed logically as one centralized unit, and the ACID properties are guaranteed via the two-phase commit protocol.

In IoT systems, the picture is dramatically different, with a massive—and growing—number of data sources; sensors, RFIDs, embedded systems, and mobile devices. Contrary to the occasional updates and queries submitted to traditional DBMSs, data is streaming constantly from a multitude of “Things” to IoT data stores, and queries are more frequent and with more versatile needs. Hierarchical data reporting and aggregation may be needed for scalability guarantees as well as to enable more prompt processing functionality. The strict relational database schema and the relational normalization practice may be relaxed in favour of more unstructured and flexible forms that adapt to the diverse data types and sophisticated queries. Although distributed DataBase Management Systems (DBMSs) optimize queries based on communication considerations, optimizers base their decisions on fixed and well-defined schemas. This may not be the case in IoT, where new data sources and streaming, localized data create a highly dynamic environment for query optimizers. Striving to guarantee the transparency requirements imposed in distributed DBMSs on IoT data management systems is challenging, if not impossible. Furthermore, transparency may not even be required in IoT, because innovative applications and services may require location and context awareness. Maintaining ACID properties in bounded IoT spaces (subsystems) while executing transactions can be managed, but is challenging for the more globalized space [4]. However, the element of mobile data sources and how their generated data can be incorporated into the already established data space is a novel challenge that is yet to be addressed by IoT data management systems.

The following section provides details of some data management solutions for IoT that integrate all or part of the above mechanisms. These proposals are then analyzed in light of a set of design primitives that we deem necessary for a comprehensive data management solution for IoT.

## 3. Analysis of IoT Data Managements Proposals Against Design Primitives

**Figure 2.**
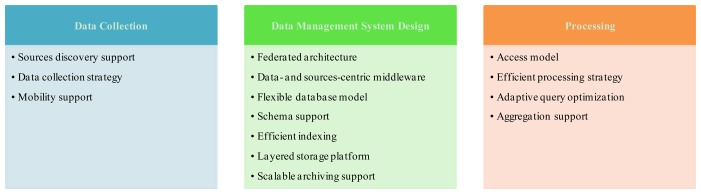
Design primitives for an IoT data management solution.

There are a number of design primitives that determine the logical and physical structure of data management solutions for IoT. Addressing all of these design primitives—illustrated in Figure 2—is essential in building a comprehensive IoT data management solution. These design primitives are organized into three main dimensions: data collection, data management system design, and processing. Data collection elements target the discovery and identification of “Things” and subsystems—static or mobile—whose data is to be fed to the IoT data stores. Data management system design elements address the architecture of the data management system and how data is to be stored and archived. Finally, processing elements deal with the actual access to data stores.

### 3.1. Data Collection Elements

*Sources discovery support*: One of the value-added services that IoT is projected to provide is the ability to tap into diverse sources of data that may not necessarily belong to the same IoT subsystem. Therefore, a sources discovery mechanism is needed for IoT applications so that they can announce their service needs and get responses from sources whose data can satisfy these needs. Alternatively, sources can periodically announce their services; the data they can report and produce. An example framework that addresses the discovery of data sources as an integral part of data management is proposed in [11]. The system may discover data sources either via crawling, or via starting with a predefined set of data sources that may later grow as new ones are discovered. Energy-efficient solutions for managing data from mobile data sources were outlined in [12]. Position prediction was used to lower the energy consumption of position tracking technologies, and context monitoring was proposed to reduce unnecessary computations and communication.

*Data collection strategy*: The collection of data from the “Things” layer may be temporal or modal. Temporal data collection involves collecting data from all “Things” at specified intervals, while modal collection involves collecting data pertaining to specific elements. The variation in data needs that is to be expected in IoT systems may dictate having more than one database schema to accommodate the two data collection methodologies [13].

*Mobility support*: Mobile devices and entities are a prominent subset of the “Things” in IoT. As they move, they need to still be able to report (and access) data to data stores in a transparent way. Solutions have been proposed for database managements systems that run on mobile devices, which use a session-based synchronization system for data exchange, and store-and-forward mechanisms to facilitate data synchronization [14–16]. Publisher/subscriber-based systems have been proposed for notification-based data delivery from and to mobile devices [17] with considerations for intermittent connectivity [18] and transactions processing [19]. Implementations for such systems are in place for the vehicular [20] and WSN [21] IoT subspaces. However, no clear proposals have been found in the literature that support mobile devices acting as data sources to continuously report/update data to centralized databases, a scenario that would be prominent in IoT. Connectivity abstraction and maintaining the semantic context on the move are the main factors that need to be addressed in order to support mobility in IoT data management.

### 3.2. Database System Design Elements

*Federated architecture*: Concepts from distributed and federated database systems can be adapted to the needs of IoT data management. Distributed database systems manage a single database distributed over multiple sites. Federated database systems, on the other hand, manage independent and possibly heterogeneous data stores at multiple sites. In a federated architecture, a site's full autonomy over its data is maintained when it chooses to participate in a database federation, based on that site's operational requirements [10]. If the different subsystems forming IoT are to be viewed as independent “data factories” with autonomous database systems, then it would be interesting to explore the adaptation of query optimization targeted at distributed heterogeneous database systems [22] to the inherently distributed and heterogeneous IoT subsystems.

In addition to federated architectures, there is an increasing trend to build IoT systems around service-oriented architectures [23,24]. Deploying architectures that merge physical “Things” with virtual services requires rethinking communication- and storage-intensive database operations, such as joins and aggregation, to provide interoperability features. Furthermore, novel techniques are needed to join real-time spatio-temporal data residing in lower layers of the IoT network with historical data stored at database servers in higher layers. This may prove especially beneficial in e-Health applications for example; where a patient's historical data can be integrated with their real-time status reports to construct a patient's history.

*Data- and sources-centric IoT middleware*: There is a need for data-centric middleware between the communication-centric “things” network and the storage-centric data stores. This middleware layer will serve to provide more versatile capabilities for discovering data sources and accessing and analyzing heterogeneous and geographically distributed data [25]. The middleware layer will also target the scalable transfer of large data volume sent from the network to the data stores. There is a need to leverage the full potential of knowledge that can be harnessed from different data sources within IoT by providing the means to discover such sources based on location, modality (*i.e.*, source type) or global query needs that are agnostic to the underlying network.

An architecture that incorporates middleware for data processing in large-scale WSNs to mask network heterogeneity and facilitate data aggregation is proposed in [26]. The core element in this architecture is a virtual sensor that integrates multiple data streams from real sensors in lower layers into a single data stream. Input stream queries are evaluated and the results are stored in temporary relations, and the result of combining these streams is stored permanently only if required, otherwise it is consumed by the application.

*Flexible database model*: In relational database systems, the principles of atomicity, functional dependency, and normalization are used to define the structure of the relations in a relational database. Database models that depart from the relational model in favor of a more flexible database structure are gaining considerable popularity, although it has been shown that parallel relational DBMSs outperform unstructured DBMS paradigms [27].

*Schema support*: Database schema formally defines the structure of a database system. In the relational model, schema is defined beforehand as tables and relationships linking those tables, and all data insertions/updates must adhere to that schema. In recent data management solutions that support large and diverse data volumes, it has been customary to go for non-schema solutions to allow for a more flexible structure of the database [28]. There are a number of trade-offs between enforcing a schema and having a non-schema solution. The lack of schema leads to records being parsed at run time, as opposed to load-time parsing that is typical in relational database systems. This causes degradation in performance for non-schema systems, as compression becomes less effective. The lack of schema will also lead to users writing their own parsers, which compromises the interoperability that is desirable for IoT applications. Query optimization is challenging in non-schema systems, because of the lack of knowledge about indices, table partitions and cardinalities, and statistics about data values [27].

*Efficient indexing*: Indexing is heavily affected by insertion operations and is a primary design element for storage systems. Having multiple indices that are used for different retrieval requirements is essential for the dynamic IoT applications. However, building and storing indexes to facilitate data access and retrieval for the massive volume of streaming sensors data can be prohibitively costly both in terms of the extra storage required and the processing overhead (e.g., slower insertions). Therefore, trade-offs between efficient retrieval and scalable index storage need to be made when developing indexing techniques. Furthermore, dynamic spatio-temporal indexing needs to be adapted for streaming data. Models to achieve this dynamic indexing include sliding windows indexing, multi-granule indexing, wave indices, and the time index model [29].

In [30], dynamic indexing is proposed only for frequently queried data. Intelligent aging mechanisms are used to handle the limited storage space at the sensors, with emphasis on the least valuable or least accessed data. In addition, aggregation and summaries of data can be used to further optimize storage capacity. Proxies that perform intelligent spatio-temporal caching and indexing of results summaries are used to logically unify query views from heterogeneous sensor platforms.

*Layered storage platform*: Contrary to traditional database systems, where “persistent” data is collected *a priori* and stored once in centralized or distributed database servers, IoT data is continuously updated as the statuses of things and monitored phenomena change. Even if it was possible for all of the data generated by things at a certain point in time to be stored in permanent storage, it will quickly become obsolete and new data will become available. Stream and real-time databases are not new paradigms [31,32], and their principles were used for environmental sensor networks [33]. However, the design of such systems addresses centralized storage locations, and is not suitable for the scale and distribution of data volumes generated by IoT sources, which requires more than efficient update and access mechanisms [34,35].

Two approaches can be adopted to address the issue of storage locations for IoT data: having each of the systems that make up the IoT use its own dedicated database system, and storing data at the memory units in the objects that generate the data and treat them as a decentralized database. In the first approach, raw or partially aggregated data is moved to aggregation points and mass storage facilities within an IoT subsystem. Having dedicated storage facilities for the different systems forming the IoT poses a number of challenges. Database access issues such as query/transaction processing and concurrency control will need to adopt mechanisms to discover IoT systems with storage facilities, since it is not feasible to provide a finite list of all the systems linked to the IoT infrastructure. The existence of privately-owned IoT systems will dictate a need for credentials management and seamless access permissions. The heavy dependence on the transmission of streaming data from sources to storage facilities will result in high communication costs. This is made more complicated by the need for different communication technologies for different object classes and transmission scenarios.

As opposed to the traditional centralized storage (centralized is meant here as a figure of speech, indicating server-based storage systems; server clusters and distributed storage systems are assumed for large data volumes), emerging trends opt for storing the data as near as possible to its production points—at the objects' memory units [30,36]. This eliminates the need for communications infrastructure to transmit raw data to centralized storage facilities and opts for transmitting only the results if needed. This also leads to enhancements in the timeliness of query processing and response. However, this direction has a number of performance issues that make its full adoption a challenge. The most important of these challenges is the unavailability of scalable storage units in all of the objects involved. Devices have varying capabilities in terms of processing, energy profiles, and storage, with the majority having resource constraints that have led to design optimizations such as the migration of sophisticated processing tasks to upper layers where more powerful devices reside and the miniaturization which allows for very limited storage space. Each device category will have to add metadata describing its data formats for later query optimization and response, which means that query processing and optimization techniques have to adapt to a wide range of data formats that may not be known fully beforehand. Furthermore, the data stored at the things layer will have to be transient; newly generated data will have to replace older data items to accommodate the limited storage capacities. This will limit logging and archiving functions that may be needed later for trend analysis. Trade-offs between centralized and local storage will be subject to the specific application's needs. To address the scalable storage limitations that may result from the increasingly large data volume generated by the “Things”, cloud migration is an emerging trend that is being proposed as a solution [37,38]. Cloud platforms provide an excellent opportunity for data migration from privately-owned servers. This will result in fewer server facilities and a more optimized utilization of energy resources. On the other hand, it should be noted that moving to the cloud will result in increased communication and processing overhead as the cloud becomes smarter.

The work in [30] proposes to move from classical view of sensor data as streams that are continuously filtered and aggregated to a storage-centric view in which sensors are equipped with flash memories and embedded platforms to store data locally at the nodes, thus contributing to reduced communication overhead as query processing migrates to the nodes. A database management system, StoneDB, considers the locally stored sensors data to form a database that can support archival queries as well as mining tasks. Transmission takes place only to communicate queries to the sensory database and results back to the query initiator.

The work in [39] proposes the usage of a customizable data management infrastructure that can be tailored according to the varying requirements of WSNs. Special focus is given to supporting robustness and reliability to handle node failure and unreliable communication, via the use of RAID-inspired distributed data storage in the form of a data storage layer that resides between a WSN and the customizable DBMS. Different roles are used for different data management needs; *endpoint sensor* storage for storage of simple key-value pairs representing sensor measurements, *cluster gateway* with SQL support and buffer management for querying WSN data, and *data aggregator* with advanced data management features such as indexing, buffer management, and possibly security for storage and query processing.

*Scalable archiving*: Scalable, adaptive, heterogeneous archiving mechanisms are needed for the vast data volumes of IoT, with efficient discovery facilities for retrieval and analysis purposes varying degrees of retrieval frequency. Data expiry schemes need to adapt “usefulness” metrics instead of simple time-centric metrics.

### 3.3. Processing Elements

*Access model*: In order to access data, querying languages have been used for relational systems, and later adapted to sensor networks. Structured Query Language (SQL) has been the de facto standard for data access, with standard selection/projection/join/aggregation operations that can be nested for complex queries. Additional constructs have been added to the language in order to accommodate new forms of data, such as TinySQL for sensor networks [40] and StreamSQL for stream processing [41]. As SQL has become too complex due to the continuous extensions as new capabilities are added, developers for the various applications envisioned for IoT will find it hard to learn all of SQL's dialects and tricks while they may need only a subset of them. Therefore, it has been suggested that a more flexible form be used, in which an SQL dialect or predefined configuration is chosen according to the specific requirements of the scenario at hand [42]. Moreover, a shift from modular programming to feature-oriented programming is proposed to customize software that is used to access databases. This enables the development of customizable data management depending on the underlying system's desired features [43,44].

*Efficient processing strategy*: The two main purposes of collecting data from the “Things” layer in IoT systems are reporting and analysis. Both involve the processing of data at some point in the system in order to aggregate/infer useful information. Determining the location at which data is to be processed in IoT systems is a primary design concern that should take into consideration the decentralized nature of the system and the volume of data produced. Two processing approaches can be deployed for IoT systems: in-network processing and centralized processing. In-network processing involves moving the “program” down to the data and sending only the results back to the user(s), thereby reducing the volume of data that needs to be transported to centralized storage at upper architectural layers in the system. Centralized processing, on the other hand, requires that data—either in its raw form or in an aggregated, more compact form—be transported to persistent storage to enable sophisticated analysis tasks. A hybrid of both techniques can be used for a more flexible processing mode, with varying degrees of customization to accommodate the diverse IoT application needs.

*Adaptive query processing and optimization*: Query processing is traditionally performed near data stores in order to provide query execution plans, which are basically data-fetching plans. Traditional query optimization involves assigning a cost to each of the different plans for obtaining data in order to choose the plan that is least costly. In the context of IoT, query processing additionally involves finding plans to fetch data from sensors and devices that are geographically distributed and returning aggregate readings as results. Therefore, it requires a large volume of message exchanges that translate into communication overhead. Lowering this overhead involves migrating query processing closer to the lower layer of things, and adopting spatio-temporal optimization to account for localized queries. Query optimization for WSNs has been extensively researched in literature since WSNs are the most prominent subsystem of IoT; an in-depth review can be found in [36].

Query optimization that involves choosing the best query plans based on their respective power costs are explored for WSNs in [45] and database servers in [46]. In [45], sensing and routing data and metadata collection are incorporated into a query optimizer in order to find the best query execution plan with the least energy costs. However, optimization is done for a single query at a time and does not take into account the dynamicity of the network. In [46], basic database operations such as read/write and join operations are assigned power profiles, and a query plan is assigned a power cost as a function of the operations used to execute that query plan. Adapting a similar approach for IoT queries will involve knowledge of the power profiles of intermediate nodes and lower level sensors in order to achieve the full potential of power-optimized query plans for queries that are not limited to data residing offline of servers.

Energy-efficient optimization of multiple queries is explored in ecoDB [47] for distributed large scale database systems. Batch query processing leverages the presence of common components in multiple queries within a processing workload. This is achieved by queuing queries and not executing them immediately. Common sub-expressions that may be present in the queued queries are then merged and executed together. This multi-query optimization achieves savings in energy consumption at the expense of an increased average response time resulting from the explicit delays.

Multi-query optimization was also used for energy-efficient query processing in WSNs in [48]. Two tiers are used to optimize the execution of queries: A base-station tier rewrites a set of queries to produce a synthetic set of queries with redundancies removed and common sub-queries merged. A number of in-network optimizations are then used to optimize the transmission of the query-set results. Three techniques are used to achieve in-network optimization: time sharing between temporal range queries, sharing common readings among similar queries via readings broadcast, and aggregation of results from the sensors responding to the queries to the base-station which has sent the synthetic queries.

*Aggregation support*: The continuously changing nature of data produced by smart objects makes it unattainable to invest in bulk data collection and storage at one repository as is the case in traditional DBMSs. By the time this is done, data of time-sensitive nature will have expired. Therefore, it is desirable to allow for data storage to take place progressively in real time as data is generated. However, this creates the challenge of transmitting large and streaming volume of data to storage facilities. Aggregation and fusion of sensor data is essential for lowering the communication overhead expected from transmitting raw streaming data. In addition, aggregation and fusion may be requirements for certain applications where raw data storage has no added value. One factor that needs to be taken into consideration is the potential loss of accuracy resulting from dropping underlying detailed data. Therefore, intelligent aggregation should be a design factor for IoT data management.

Energy-efficient aggregation mechanisms that focus on approximate aggregates for Wireless Sensor Networks (WSNs) data are proposed in [49]. Uniform sampling with sample averages is assumed to represent real data averages. Flow-based partial aggregates are distributed among peer nodes that report to the same parent, and the partial aggregate thus moves up the flow until it reaches the query originator. Such technique may be sensitive to packet loss, therefore trade-offs between the rate of aggregation and the communication overhead is needed. Extending the aggregation process into a full implementation of application functionalities on the sensor nodes themselves has been proposed in [50], where an application's operations are decomposed into all exhaustive plans and the plan with the best performance estimates in terms of energy consumption in terms of sensing and computations is considered for execution. There were a number of proposals that addressed the design of comprehensive data management solutions for the WSN subspaces of IoT. Cougar [51] adopts a cross-layer approach to WSNs energy-efficient data management, in which the communication layer adapts to the needs of the data management layer. Cougar treats the sensor network as a distributed database where data collection is performed using declarative queries. A central query optimizer generates efficient query plans aimed at minimizing resource usage within the network for a given query. The sensors sense data and then transmit data matching some criteria to the base-station. The amount of data transferred is further minimized by in-network processing, in which the WSN is logically organized as an aggregation tree, with each node maintaining aggregated results of nodes in the sub-tree of which it is root. Optimal organization of such a tree is query-dependent, and therefore may not be practical for the large-scale IoT network.

In [52], an architecture based on distributed in-network query processing is discussed. Users input SQL-like queries at the server end describing the data needed and how it should be fused. Queries view data produced by a sensor as a virtual table with one column per sensor type. Tables are continuous streams of values, with periodic time-bounded buffering used for querying purposes. Sensors push data to designated *view nodes* where queries can pull the data or data is pushed further to higher levels in the network. Power consumption is controlled via defining a lifetime attribute for the query, thus giving the user control over the data sampling rate. Intermediate nodes are used to execute partial aggregation and packet merging to lower the communication overhead incurred by the relay of query results. The work is further extended in [40], which proposes TinyDB; a DBMS designed and tailored for the needs of resource-constrained WSN nodes, with a query language specifically designed for querying WSNs. Sensors can determine the time, frequency, and order in which to acquire samples needed to answer queries to achieve minimized energy costs. There are examples for DBMSs customized for subsystems of IoT other than sensor networks. For example, PicoDBMS is a DBMS platform supporting data management functionalities for smartcards [53], while DELite is a data management system that is designed for embedded devices [15]. Both propose special storage mechanisms and query processing customized for the distinct nature of smartcards and embedded systems respectively. However, these solutions rely on the availability of energy supplies from hosting devices (e.g. card readers) and do not address energy efficiency requirements. Based on discussions above, we can summarize the existing data management solutions for IoT or IoT subspaces relative to whether and how they fulfill the aforementioned design primitives, as shown in Table 1.

## 4. Data Management Framework for IoT

Most of the current data management proposals are targeted to WSNs, which are only a subset of the global IoT space, and therefore do not explicitly address the more sophisticated architectural characteristics of IoT. WSNs are a mature networking paradigm whose data management solutions revolve mainly around in-network data processing and optimization. Sensors are mostly of stationary, resource-constrained nature, which does not facilitate sophisticated analysis and services. The main focus in WSN-based data management solutions is to harvest real-time data promptly for quick decision making, with limited permanent storage capacities for long-term usage. This represents only a subset of the more versatile IoT system, which aims at harnessing the data available from a variety of sources; stationary and mobile, smart and embedded, resource-constrained and resource-rich, real-time and archival. The main focus of IoT-based data management therefore extends the provisions made for WSNs to add provisions of a seamless way to tap into the volumes of heterogeneous data in order to find interesting global patterns and strategic opportunities.

Some of the current proposals provide abstractions to support the integration of data from heterogeneous networks, thus paving the way for adaptation and seamless integration of other IoT subsystems. Solid and comprehensive data management solutions that support interoperability between diverse subsystems and integrate the overall lifecycle of data management with the presence of mobile objects and context-awareness requirements are yet to be developed. We propose a framework for IoT data management that is more compatible with the IoT data lifecycle and addresses the design primitives discussed earlier. The proposed framework has a layered approach that centers on data- and sources-centric middleware, and borrows the concepts of federated database management systems to guarantee the autonomy of independent IoT sub-spaces as well as flexible join/leave architecture.

### 4.1. Framework Description

**Figure 3.**
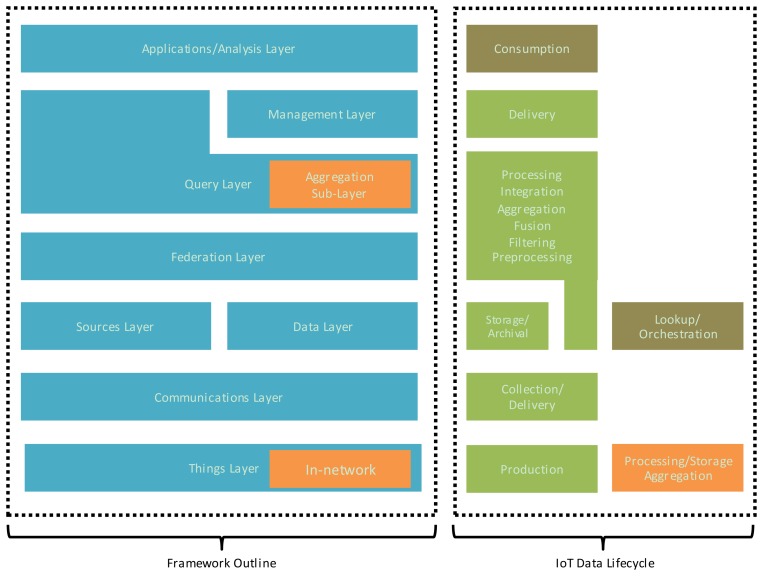
Outline of the proposed IoT data management framework and mapping of its layers to the IoT data lifecycle.

The proposed IoT data management framework consists of six stacked layers, two of which include sub-layers and complementary or twin layers. The framework layers map closely to the phases of the IoT data lifecycle described in Section 2, as shown in Figure 3, with lookup/orchestration considered to be an added process that is not strictly a part of the data lifecycle. The *“Things” Layer* encompasses IoT sensors and smart objects (data production objects), as well as modules for in-network processing and data collection/real-time aggregation (processing, aggregation). The *Communication Layer* provides support for transmission of requests, queries, data, and results (collection and delivery). The *Data/Sources twin layers* respectively handle the discovery and cataloguing of data sources and the storage and indexing of collected data (data storage/archival). The Data Layer also handles data and query processing for local, autonomous data repository sites (filtering, preprocessing, processing). The *Federation Layer* provides the abstraction and integration of data repositories that is necessary for global query/analysis requests, using metadata stored in the Data Sources layer to support real-time integration of sources as well as location-centric requests (preprocessing, integration, fusion). The *Query Layer* handles the details of query processing and optimization in cooperation with the *Federation Layer* as well as the complementary *Transactions Layer* (processing, delivery).

**Figure 4.**
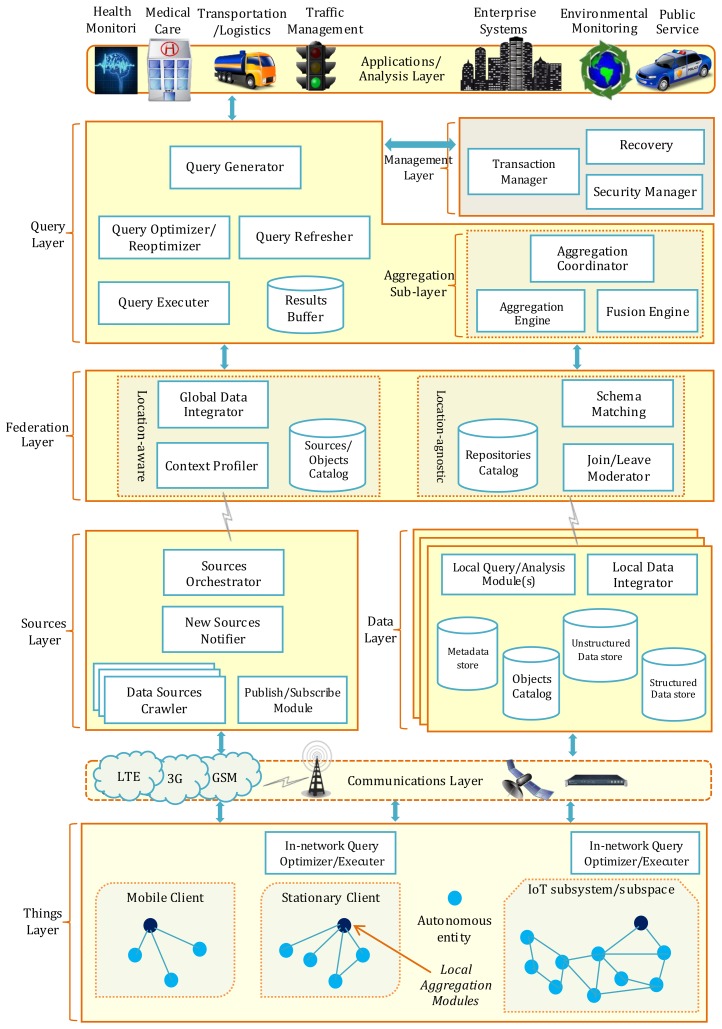
IoT data management framework.

The Query Layer includes the *Aggregation Sub-Layer*, which handles the aggregation and fusion queries that involve an array of data sources/sites (aggregation/fusion). The *Application/Analysis Layer* is the requester of data/analysis needs and the consumer of data and analysis results. The layers of the proposed IoT data management framework and their respective functional modules are illustrated in Figure 4, and discussed in the following subsections.

#### 4.1.1. Data Layer

We start the framework description with the data layer since it is the core element in data management. Understanding where and how data is stored is essential to subsequent updates, queries, and access to the data. There are two main issues to be addressed in IoT data management with regard to the data itself: the placement of storage facilities (the where), and the format to be used for data storage (the how). In our framework, we opt for a hybrid approach to data storage—with temporal, real-time data stored near at the objects' generating this data, and persistent, long-term data that is to be used for analysis catalogued and stored at dedicated facilities—is expected to yield a beneficial trade-off between the costs of storage space and data transmission on one hand and the availability of data for sophisticated analysis and queries on the other hand. The data layer is concerned with the storage of persistent data, while the things layer is concerned with the storage of transient data, among other things to be discussed at later sections.

Which data should go where is a design element that is subject to the nature of data and the requirements of applications that will have the most access to the IoT system that will utilize this data the most. Due to the global nature of IoT, data is generated at different locations that may be far apart geographically, requiring a federated approach to storage and access of real-time data. Federated data management involves the autonomous control of local sites on their data, with optional participation in the data federation for query processing purposes and only when resources allow for such participation. A similar approach can be followed with IoT data management, with data generated by the different sub-systems or groups of related objects placed at locations designated by the owners of such sub-systems. The different sub-systems can then participate in a database federation if they find their data relevant to a query, and only when their energy-constrained resources allow. The data layer therefore can be viewed as an abstract representation of the union of the data residing on the different IoT sites, with modules to locally handle this data, and with catalogs to identify the specifications of this data for the purposes of later integration into the federation at the federation layer. The data layer modules have functionalities that are similar to their counterparts in upper layers. *The Local Query/Analysis Module* performs the same functions of the query processing and optimization modules to be discussed later in the query layer, but on the level of a local IoT site. *The Local Data Integrator Module* performs simple integration processes on data that generated within an autonomous IoT system, with possibly structured and unstructured data to be unified for query and analysis results.

At the Data Layer catalogs, information necessary to access data and define sources (*i.e.*, objects or “Things”) is stored. Semantic metadata can be used to describe how data can be accessed and linked in order to facilitate efficient queries, personalization of results, and heterogeneous data integration [54]. Furthermore, data can be associated with geo-location and time tags to facilitate with semantic relevance as well as location and time context establishment; certain data is only relevant in specific locations and during specific time intervals. Defining data sources in the catalog can take a hierarchical form that resembles the semantic structure of the sources, with inter-links to represent existing dependencies. In addition, sources may be associated with confidence degrees in the accuracy level of the data they generate, which can be used for query optimization. Catalog information will be the primary source that is used to provide schema mapping for interoperability between heterogeneous data stores. Domain-specific descriptions should be provided as an integral part of metadata, also for interoperability purposes.

#### 4.1.2. “Things” Layer

The things layer encompasses the entities that generate data for the IoT system as well as modules that perform in-network, real-time processes whose results are to be transmitted further up the system. Entities can be sensors, RFID tags, mobile devices, laptops, embedded chips, vehicles, ships, and any apparatus that has embedded intelligent devices with communication capabilities. Entities can be looked at as collections or what can be called virtual entities [5]; a body area network linked to a patient, a vehicle with a multitude of intelligent devices, or an environmental sensor network. Each of these objects, whether operating autonomously or as a part of an interlinked network system, is considered a data source for the IoT network.

In order to have access to data at the things layer, there needs to be a mechanism to uniquely identify the data sources. Due to the global nature of IoT, location-based identification is pivotal to the efficient retrieval and querying of data generated by the geographically distributed things. Furthermore, modal identification should be incorporated as well to identify entities of the same type that provide the same type of data. This is similar to data-centric naming mechanisms that are followed in some sensor networks. Platform-dependent identification can be cross-referenced with location-based identification, and is used to identify entities belonging to a specific platform or entity and serving a generic purpose pertaining to that entity or platform, as is the case with body area networks linked to a certain patient. Therefore, this unique identification is tightly attached to the IoT objects and can be promptly accessed at the things layer, either by in-network processes or by crawling agents at upper layers in the framework that need to identify certain objects that may satisfy certain requests.

Two modules are of importance at the things layer and are similar in functionality to other modules in upper layers, although at a less sophisticated complexity level: The local aggregation modules and the in-network query optimizers.

*Local Aggregation Modules*: To optimize transmission and storage costs, the aggregation modules at the Things layer are deployed to provide summaries and aggregates of data whose detailed values are of limited importance and can be discarded. Since the aggregation function aims at minimizing communication costs, the aggregation points are deployed closer to the data sources. The modules collect and summarize data from multiple objects/Things that can be either homogeneous or belonging to a single system. The use of such aggregation points is subject to the processing and efficiency needs of the underlying subsystem/entity, and should not cause delays that may compromise the system's real-time performance [55].

*In-network Query Optimizers:* Another module that is to be deployed at the “Things” layer is the in-network query optimization/execution module. This module will be activated for real-time queries that need to be executed at the network level and for which only end results are needed in upper layers. The data that will supply such queries with results may still be reported periodically to permanent storage at the upper data layer, but are processed in real-time to provide prompt reporting for delay-sensitive queries.

#### 4.1.3. Communication Layer

The communication layer connects the distributed data sources to more concentrated data storage and processing units. Inter-objects as well as objects-to-infrastructure communication technologies are to be used, and interoperation guarantees are provided at upper layers. Communication takes place also close to the federation layer as multiple geographically dispersed data repositories can be engaged for sophisticated query and analysis purposes.

#### 4.1.4. Sources Layer

In distributed database systems, database fragments are stored in predefined and finite locations. The system design dictates that metadata store the locations of database fragments beforehand for querying and update purposes. The situation differs drastically for IoT data, where sources that will generate and deliver data are (1) distributed over diverse locations that vary from dedicated objects to implants on objects; (2) not finite, with new sources becoming available continuously; and (3) autonomous, with no unification of schema or definition of metadata. These characteristics make it challenging to execute real-time queries placed to the things layers if there is no way to identify which sources or sub-systems can respond to the requests. There needs to be a layer on top of the things layer that handles the seamless and transparent identification and unification of data sources for query processing purposes. This layer is mainly targeted at the requests on the downlink, and is not concerned with the periodical reporting of generated data to upper layers for storage and archiving purposes.

*Sources Orchestrator*: The sources orchestrator is concerned with translating a query execution plan in terms of the sources that should be involved in executing that query plan. It works closely with the query optimizer to find the sources that match the query requested data and tweak the execution plan so that it becomes location-aware. In addition, it builds the plan of cooperation and hierarchy of results delivery that is to be followed by the data sources.

*Sources Crawler(s):* The Internet of Things has a flexible architecture that accommodates new sub-systems as they are installed and activated. If seamless and scalable merging of such systems into the overall architecture is to be supported, their existence should be made seen by the system as a whole. This can be done proactively, by having these systems announce their existence to the IoT infrastructure; or reactively, by running discovery routines whenever new queries are placed to the things layer. Proactive appending of new sources is discussed in the next module. Reactive discovery of new sources is done by either having the crawler do periodical scans or do scans only upon reception of data query requests.

*New Sources Notifier:* As new sources are found by the sources crawler, there needs to be a notification mechanism through which real-time, continuously updated queries become aware of the existence of such sources. In addition, the data specifications of these new sources need to be reported to the data layer in order to be merged with the objects catalog and metadata store. The new sources notifier can also be used to proactively alert the IoT infrastructure about the specifications of new sub-systems or devices so their metadata can be included in the system for future reference and access. New sub-systems or devices make themselves known to the infrastructure by directly reporting their specifications to the new sources notifier, which in turn verifies the authenticity of these reports and passes the specifications to the objects catalog and metadata store that is either closest geographically or most related semantically.

*Publish/Subscribe Module:* The publish/subscribe module acts as a broker between the “Things” layer and the federation layer, accepting announcements of presence by “Things” (sources of data) and queries (data consumers). Things publish descriptions of the data/services they can provide to the module, and the module can engage their active participation subsequently in queries.

#### 4.1.5. Federation Layer

The federation layer lies at the center of the framework, and provides the glue that joins dispersed IoT subsystems and data sources together to form a globalized view of the IoT system. It provides interoperability features for the diverse data types and repositories that are to be joined together to answer a specific query. Location-agnostic modules enable the different sources/subsystems to seamlessly join and leave the federation, while location-aware modules handle queries that have explicit location-specific requirements.

*Repositories/Sources Catalogs:* The repositories catalog holds metadata about the different data repositories/databases that wish to participate in the IoT data network. The sources catalog is a more scalar version of the repositories catalog, in which descriptions of the objects that provide data are defined. Metadata such as location, time of joining/leaving the network, and the specifications of the data included are typical in such catalogs.

*Join/Leave Moderator*: The join/leave moderator supports the federation principle in this framework. It manages the inclusion/exclusion of data repositories that announce their participation in/withdrawal from the provision of data for queries or analysis tasks. Repositories that wish to be data providers for the IoT infrastructure declare this to the join/leave moderator, with their data schemas or specifications and desired performance and security parameters. The moderator adds the repositories' descriptive information accordingly to the catalog. Repositories can nevertheless be inactive intermittently if their local administrative needs are set to have higher priority and cannot accommodate participation at global views. At any point in time, the join/leave moderator knows which repositories are actively available for participation in query/analysis tasks, either via periodic announcements or via on-demand probing. The moderator can then negotiate participation in a specific query or analysis task based on the repositories' local workloads, processing priorities, and availability of the needed data. Repositories can announce their desire to permanently deactivate their participation in the IoT data federation so as not to be contacted for future query negotiations by the moderator.

*Schema Matching Module*: The schema matching module should provide a unified and coherent view of the diverse and heterogeneous data repositories/databases that are accessed by the federation layer. The same piece of data can be available under different names at different repositories, and similar entities or classes may be represented differently in the varying databases' schemas. Therefore, schema matching may depend on comparing attribute values or defining ontologies for data attributes in order to mask semantic heterogeneity and provide a seamless schematic view of data. Since matching the schemas in the presence of a dynamic data repositories environment such as the one characteristic of IoT, it is not practical to define a unified global schema in advance. A more convenient alternative is to perform dynamic on-the-spot data-level matching whenever repositories are engaged to respond to query/analysis tasks. Schema-level matching based on schema information can be predefined for core attributes and object classes to support data-level matching.

*Context profiler*: A context profiler will provide context specification support for queries coming from upper layers with no/little information about the location of the data of interest. The purpose of queries placed directly at the “Things” layer is to collect real-time information concerning certain phenomena or locations. The context profiler should determine for each such query the location of the data sources that have the most potential of holding results for that query. Although the main contextual support provided is geographic, other context specifications such as time frames or modalities can be supported as well. An example query with multiple context specifications is a query that may reach the data sources layer asking for identities of patients (modality) within a certain city (location) who have shown certain symptoms (query constraints) that are indicative of a quickly spreading (time frame) epidemic.

*Data Integrator:* The data integrator closely collaborates with the query layer to facilitate access to heterogeneous data via processing the information included in the sources catalog. The data integrator will assist in the execution of semantic queries that do not state the repositories from which to fetch data, but rather provide location or time constraints for data sources. It will also map the different data formats/semantics into a unified form that is suitable for query results that are to be provided to upper layers. The data integrator will work as well to provide seamless results format for queries that join both data from well-defined repositories with real-time data from dynamic data sources. The data integrator is deployed at the data layer as well to provide similar adaptation facilities to local IoT subsystems/sites for local query processing.

#### 4.1.6. Query Layer

The query layer encapsulates the elements necessary for generating, optimizing, and executing queries on the IoT database. It is deployed both at the federated and local levels; the local level being that governing the subsystems deployed by individual organizations or agencies. This way, a global view of IoT data can be obtained while localized data views can still be generated by the underlying systems forming the IoT infrastructure.

*Query Plan Generator*: The query plan generator takes as input the specifications of the desired output, either from the application or directly from the user, and transforms it into a query written in a standard query language format. Possibly multiple query plans are then generated either as command trees or as sets of steps; query representations that conform to the data entities defined in the database schema and detail the instructions of how to fetch the needed data. Semantic and context queries that dictate only the desired location(s), time interval, or business-specific semantic requirements, need also to be transformed into executable plans. This will be done in collaboration with the data adapter. The plans for a query are then passed to the query optimizer to choose the best plan to execute.

*Query Optimizer/Reoptimizer*: The query optimizer receives a set of query plans for a query, and finds the plan that is most efficient in executing the query. Each plan is assigned a cost that is estimated based on evaluation criteria that are either predefined in a data dictionary or fed at runtime by the user submitting the query. Example evaluation criteria are the number of input/output operations, processing time, delay constraints, and temporal/spatial/modal constraints for time-series/location-aware/source-specific data. The optimizer can “reoptimize” the execution of queries that are targeted at dynamic data sources, if required by the user posting the query. Optimization is reinitiated to incorporate/exclude sources whenever new sources become available or sources become no longer available to provide results to the query.

*Query Refresher*: The query refresher is activated for queries whose results are streaming data of periodically generated or updated nature, or whose sources dynamically join/leave the network. Dynamic real-time updates to query results need to account for the storage space allotted to the results. This can be done either by summarizing results into synopses, by gracefully aging results according to some relevance requirements, or by providing approximations to query results with guarantees on the approximation accuracy in representing the actual data. A temporary storage buffer for results can be used to hold the step-by-step intermediate results of the query as it gets executed. It can also hold the streaming results from updatable queries as more data is received from the “Things” layer.

#### 4.1.7. Aggregation Sub-Layer

IoT data repositories are globally distributed over geographic areas and hold massive volumes of data that is constantly generated and may change as time progresses. Contrary to aggregation close to the “Things” layer—which involves basic summaries and aggregates for real-time queries—sophisticated aggregation provisions are needed close to the federation layer, where applications have access to both real-time and stored data.

*Aggregation Engine:* The aggregation engine is engaged according to query needs, and works closer to the repositories in order to perform summary operations of data based any number of criteria such as time, location, or modal context. It works similarly to the aggregation module that is placed closer to the “Things” layer, and should be orchestrated by the query optimizer in order to further lower the data volumes to be sent from repositories.

*Fusion Engine*: In addition to aggregation, which provides various forms of summaries for data, fusion functions should be provided at this layer as well to support more sophisticated data merging capabilities. Fusion engines placement is similar to aggregation engines placement, although the fusion techniques should be as simple and efficient as possible, and used mostly for data whose delivery can be delay-tolerant.

*Aggregation Coordinator:* For queries that may involve accessing abstract systems or repositories at different locations, an aggregation/fusion coordinator can be used to collect the data from the involved repositories, and organize the order in which data from the different repositories are processed for summaries.

#### 4.1.8. Management Layer

The management layer is concerned with the mechanisms needed to provide access and security to the various data stores in the data layer of the framework.

*Transaction Manager*: The transaction manager handles the execution of transactions that are more related to business processes and services. Depending on the type of transaction submitted to the manager, it can deploy either a classical single-source execution mechanism, or deploy global or distributed execution strategies. The strict ACID properties that are required for successful transactions in order to guarantee data consistency may be relaxed in favour of the more trending eventual consistency guarantees [56].

*Recovery Manager*: The recovery manager is concerned with restoring the data repositories into the most recent consistent state after the occurrence of a failure due to electricity, crash incidents, corrupted files, *etc*. This is usually done by rolling back all the transactions or operations that were taking place within the data management system and have not yet been committed successfully. Archiving is one way to recover lost or damaged data in primary storage space, but replicas of data repositories that are updated concurrently with the primary repositories can be used for sensitive systems with strong data availability requirements. Replication can be demanding in terms of storage and may degrade performance due to if a concurrent updates strategy is enforced. However, in the case of IoT, this may not be the case due to potential “natural” replication; the availability of the same data needs from multiple sources that are already installed and maintained independently. Therefore, the recovery manager may only be concerned with independent repository maintenance via the Redo/Undo recovery mechanisms already deployed for database managements systems [8,57]. Temporal data or data residing at the “Things” layer may not need strong recovery mechanisms, unless the data is of sensitive or archival nature, although proposals to recover data stored in embedded systems exist [58].

*Security Manager:* The security manager should include data protection and privacy measures in accordance with legal frameworks that are of relevance to both the protected data and to the final users. Privacy is highly critical for IoT networks since some “Things” that generate the data can be owned by private entities or persons, such as is the case with data generated by medical devices or vehicles. Varying degrees of data privacy requirements can be defined, depending on the nature of data and the entity that generates the data [59]. Therefore, designing robust security and privacy measures that are tightly integrated with data management solutions is essential to the successful deployment of IoT.

#### 4.1.9. Applications/Analysis Layer

The primary reason for deploying a massive data storage strategy in IoT is to harness the wealth of information that can be produced from the correlation of data and to extract potentially interesting patterns/trends. This information can be of use for the proper functioning of the system, future improvements, and novel or unconventional business opportunities. The applications/analysis layer will be concerned with the utilization of the information produced by IoT via delivering a set of services and analysis capabilities to the end users of the IoT system. Analysis should be deployed at federated and local scales, to provide a global and diverse view of IoT data plane while still facilitating localized and autonomous access/utilization to the data pertinent to IoT subsystems.

Four general categories are identified as prominent application domains for IoT [60]: Transportation and logistics, healthcare, smart spaces, and personal/social applications. Most of the applications under the umbrella of these domains are data-intensive, with varying data/analysis needs according to the type of services they provide to end users and businesses. In addition, different applications may need data access and processing at the lower Things layer or the higher data layer, depending on the real-time requirements and level of complexity needed for analysis. Pattern recognition and data mining techniques can be used for the multitude of IoT applications. However, they need to take into consideration the three factors that distinguish IoT data; the increasingly large data volume, the highly unstructured and heterogeneous nature of data itself, and the geographically distributed storage facilities.

### 4.2. Comparison of the Proposed Framework to IoT Architecture Reference Model

The IoT Architecture Reference Model (IoT ARM, or IoT-A for short) is a project funded by the European Commission within the Seventh Framework Programme FP7, and aims at defining a reference for building compliant IoT architecture so that new systems and services can be created that interoperate with the IoT ecosystem's existing systems [5]. The ARM defines a reference model that is composed of a number of sub-models. The *domain model* defines the basic attributes and responsibilities of IoT devices, resources, services, and virtual entities (abstractions of physical sensors into physical devices and units; smartphones, vehicles, patients, *etc.*) and the relationships between them. The *information model* defines—on a conceptual level—the attributes and relations of the data that is handled in an IoT system, which includes modeling the information flow and storage and how they are related. The *functional model* breaks up the IoT architecture into manageable parts, defines their interrelationships, and produces functionalities that can be used to build IoT systems. The *communication model* defines the communication paradigms that connect entities within the IoT. The *trust, security, and privacy model* defines concepts related to system dependability and compliance to expected behavior, security of communications, and protection of private data and control of information disclosure within the IoT context.

Of special importance to our work are the information and functional models. Our definitions of data types when discussing the IoT data lifecycle are compliant with the IoT-A definitions of data to be either real-time, summarized or aggregated, inferred by in-depth processing, or reconciled (preprocessed and enhanced) for sophisticated data analysis. The IoT-A functional model breaks IoT functionalities into functionality groups, synonymous to the layered approach we have followed in our framework. The device functionality group maps to our things layer, with possible storage capacities made possible on the devices as we have defined in the things layer. The communication functionality group maps to our communication layer. The IoT service functionality group maps to the data and sources layer in our proposed framework, although our proposed framework details many modules that are abstracted in the IoT-A functional model. The virtual entity functionality group can be mapped to the federation layer in our framework. However, since our framework is data-centric, the virtual entities in the federation can be of a scale bigger than that which is meant by the virtual entities in the IoT-A. The federation layer extends also to the service organization functionality group, which also handles service requests from applications and businesses, and therefore can be mapped to the query layer. We merge the business process management and application functionality groups into the applications/analysis layer, but we do not detail the specifics of this layer as is done in the IoT-A, leaving this as future work. Two functionality groups in the IoT-A—management and security—are only partially addressed in our framework, and further elaboration is intended as future work.

When it comes to data handling, the IoT-A information view that stems from the information model provides a more detailed outlook as to how data is stored. IoT services provide access to data produced within the system, either real-time, raw data (on demand) or higher-level data that has been processed/aggregated. Special IoT services are defined as “history storage”, both at the sensors level and at the virtual entities level, which is analogous to the storage patterns we have included in our framework's thing layer (objects' internal storage) and data layer (repositories/archives). Unlike our use of catalogs and discovery modules to manage the discovery and indexing of data sources, the IoT-A uses service descriptions that are either provided by the IoT services themselves or by special management components in order to make the services visible and discoverable within the IoT system. A service resolution component is responsible for service discovery, which is similar to what our proposed framework provides in the sources layer. Information flow follows three exchange patterns similar to what we have defined in the IoT data lifecycle; namely, the data push, the request/response, and the subscribe/notify patterns.

### 4.3. Illustrative Use Case

An IoT-empowered mobile health (m-health) system would have wearable or implanted health monitoring sensors (devices, things) on the patient (an abstracted virtual entity), which report on the patient's vital signs and possibly track her/his whereabouts as her location changes, either indoors or outdoors (push exchange pattern). These sensors are connected wirelessly to concentration points for periodic data collection/reporting (aggregation). The patient's smartphone can be such concentration point, and it can also interact with and control the patient's surrounding environment (e.g., home/hospital apparatus, bus chairs, sensors on a sidewalk). Vital readings that are collected periodically by the smartphone are reported wirelessly to the respective caregiver's network (communication) and stored in the patient's respective health records in data repositories (data layer repositories). Emergency or abnormal events concerning the patient are also reported back to the caregiver's data repositories and the caregiver is alerted for proper assessment and response (notification exchange pattern). If the patient experiences a severe health condition while driving for example, smart vehicles can “sense” such a condition and report it to the nearest emergency unit (enabling federation of patient data and vehicle data). Information analysts at the hospital have access to the patients' information included in data repositories and can profile and assess a single patient's condition based on his history (query, application/analysis). They can also analyze the collective patients' health profiles in order to find or infer interesting patterns related to possibly spreading health conditions, such as post-op infection incidents related to operations performed at that given hospital. The scale of analysis can be further widened to include health records that are accessed via the Internet on a city-wide, state-wide, country-wide, or even world-wide scale (sources discovery, then data federation). This can serve to identify the existence of epidemics or seasonal symptoms, as well as visualize their spread rate, patients' severity levels, and locale. This can help in the prompt containment of life-threatening conditions. On a short-term scale, findings of such analysis could initiate a series of commands back to individual patients' smart environments in order to provide proper isolation and/or decontamination (transaction management as a series of commands executed as one unit). Longer-term uses of analysis findings can drive future health care policies and deployment strategies.

This example highlights a data-intensive environment in which the availability of data for large-scale analysis is pivotal in discovering valuable knowledge that can shape strategic action plans. The various levels of data availability (single patient, patients at a single hospital, patients at certain localities) are made possible in the framework since there are three levels of data collection and processing: single data source, single data site with multiple sources, and a federation of data sites/sources. In addition, the real-time and offline processing modes of data are supported via in-network processing as well as on-site/federated analysis. The system is flexible in the sense that as new sources of data become available (e.g., new patients, new hospitals, new localities), they can be seamlessly merged into the existing system, because it supports federation rather than rigid structure. It is not mandatory that a certain level of data availability be the exact aggregate of all sub-levels; analysis of data about a certain municipality, for example, does not require all data from hospitals within that municipality. Overall, the framework's separation of data and processing, and the framework's support for various levels of availability, allow for various processing needs to be addressed by different entities with varying degrees of flexibility.

## 5. Conclusions

In this paper, we discussed some of the data management solutions proposed for the Internet of Things, with a focus on the required design elements that should be addressed in order to provide a comprehensive solution. The design primitives we propose cover the three main functions of handling data; how it is collected, how it is stored, and how it is processed. The current solutions are only partial in the sense that they address data management requirements of IoT subsystems such as WSNs, and include partial subsets of the desired design primitives. To compensate for this shortage, we outlined the components of a comprehensive IoT data management framework with core data and sources layers and support for federated architecture. The framework highlights the need for two-way, cross-layered design approach that can address both real-time and archival query, analysis, and service needs. Future work involves mapping the details of the proposed framework more closely to the reference model in the IoT-A, in-depth investigation and development of a data management solution that builds upon the proposed framework, and adding considerations of data security and privacy into the framework design in compliance with the considerations that need to be addressed in the IoT dynamic and heterogeneous environment. Another dimension that the authors want to explore is the integration of heterogeneous data sources and systems within the IoT, where heterogeneity extends from the classical notion of different data types and formats to different data sources, time and geo tags, and globally distributed locations.

## Figures and Tables

**Table 1.** IoT data management solutions addressing design primitives.

	**Target System**	IoT: [11,25]	WSNs: StoneDB [30,48], Cougar [51], TinyDB [26,39,40]	Embedded Systems: DeLite [15]	Smart Cards: PicoDBMS [53]		Distributed Data Stores: ecoDB [47]		VANETs: [20]
**Data collection**	**Sources Discovery Support**	Initial set with incremental crawling results: [11]			Context monitoring + position prediction: [12]				
	**Data Collection Strategy**	Results-Up: [26,30,51]			Data-Up: [26,52]				
	**Mobility Support**	Session-based synchronization + store-and-forward: [14,15]			Publish/Subscribe: [17–21]				
**Database System Design**	**Architecture**	SOA-based: [23,24]			Middleware-based: [25,26]				
	**Use of Data/Source Middleware**	[11,25,26]							
	**Schema Support**	Non-schema: [28]				Multiple schemas: Temporal-based + modal-based schemas [13]			
	**Indexing**	Dynamic indexing of frequently accessed data: [30]				Time indexes: [52]			
	**Storage**	Embedded storage at data sources: [31,36,51]		Tiered storage: sources-gateways-repositories storage [39]				Cloud-migration: [37,38]	
	**Scalable Archiving**	Intelligent aging using least valuable/least accessed data: [30]							
**Processing**	**Access Model**	Custom SQL: TinySQL [40], StreamSQL [41]		Scenario- and feature-based: [42–44]					
	**Processing Strategy**	In-network: [30]		At virtual concentration nodes: [26]					
	**Query Optimization**	In-network: Costs based on energy, sensing, and routing requirements [40,45]		Batch-based: Common query components [47]		Tiered: in-network basic optimization + batch optimization at the base station [48]			
	**Aggregation Support**	Sampling-based: sample averages [49]		Partial aggregates: Tree-based [49,51]; Tiered [40]					
